# Quality Improvement Project to Improve the Timeliness of Care for Children With Testicular Torsion in the Emergency Department

**DOI:** 10.1097/pq9.0000000000000576

**Published:** 2022-07-18

**Authors:** Sri S. Chinta, Matthew P. Gray, Matthew Kopetsky, Shannon H. Baumer-Mouradian, Amy L. Drendel, Elizabeth Roth, Catherine C. Ferguson, Mark Nimmer, Kevin Boyd, David C. Brousseau

**Affiliations:** From the * Department of Pediatrics, Medical College of Wisconsin, Milwaukee, Wis.; † Children’s Wisconsin, Milwaukee, Wis.; ‡ Nordic Consulting, Inc, Madison, Wisconsin.; § Department of Urology, Medical College of Wisconsin, Milwaukee, Wis.; ¶ Department of Radiology, Medical College of Wisconsin, Milwaukee, Wis.

## Abstract

**Introduction:**

Testicular torsion (TT) is a urologic emergency that requires timely diagnosis and surgery. We noted variation in the door-to-detorsion times for patients with TT at our institution and our orchiectomy rate was 25.8%. We aimed to decrease the mean door-to-detorsion time from 124.6 to 114.6 minutes or less over 12 months.

**Methods:**

A multidisciplinary team of pediatric emergency medicine, radiology, urology physicians, and nurses, was formed. Our key drivers were use of Testicular Workup for Ischemia and Suspected Torsion (TWIST) score, prompt urology consultation, and efficient transfer from emergency department (ED) to operating room. Our process measures were TWIST score documentation rate and early urology consultation rate, outcome measures were door-to-detorsion time and orchiectomy rate, and balancing measure was ultrasound utilization rate. Early urology consultation occurred when the ED provider documented telephone communication with urology, immediately after placing a testicular doppler ultrasound (TDUS) order and before TDUS result.

**Results:**

Over 2 years, 45 cases of TT were diagnosed. TWIST score documentation was implemented and was sustained at 78%. This improved early urology consultations from 40% to 60%. The mean door-to-detorsion time improved from 124.6 to 114.2 minutes. There was no reduction in the orchiectomy rate or TDUS utilization rate.

**Conclusions:**

A quality improvement project to improve the timeliness of care for children with TT resulted in expedited ED care but did not impact the orchiectomy rate.

## INTRODUCTION

Testicular torsion (TT) is a critical pediatric surgical condition with an annual incidence of 4 cases per 100,000 males younger than 18 years.^1,2^ Prompt diagnosis and timely surgical intervention are necessary to prevent irreversible damage to the testicle.^3^ Detorsion performed within 6 hours of symptom onset is one of the critical determinants of testicular survival.^3,4^ Factors related to parent recognition of symptoms and hospital arrival clearly impact time to detorsion but were beyond the scope of this project. Gold et al^5^ identified “hospital” door-to-detorsion time as an independent predictor of testicular survival. They estimated that every 10 minutes of delay in the ED increased the chance of having a nonviable testis by 4.8%. Before this quality improvement project, the mean door-to-detorsion time at Children’s Wisconsin was 124.6 minutes, and the orchiectomy rate was 25.8%. Our global aim with this project was to improve the quality of care for patients with TT and reduce the orchiectomy rate.

The Testicular Workup for Ischemia and Suspected Torsion (TWIST) score is a validated clinical score (Table 1) to screen and risk-stratify patients with symptoms concerning for TT. Possible TWIST scores range from 0 to 7, with scores of 0–1, 2–4, and 5 or more, associated with low, intermediate, and high risk of TT, respectively.^6–8^ TWIST scores have not been previously reported as a tool to expedite patient care for patients with TT. We theorized that by using the TWIST score to rapidly identify patients at high risk for TT and initiate early urology consultation, we could decrease both door-to-detorsion time and orchiectomy rate. To decrease orchiectomy rate, we aimed to decrease the mean door-to-detorsion time for patients diagonised with TT from 124.6 to 114.6 minutes or less over 12 months.

**Table 1.** TWIST* Score Components and Recommendations for Patient Care

Component	Score
Testicular swelling	2
Hard testicle	2
Absent cremasteric reflex	1
Nausea/emesis	1
High-riding testicle	1
Total score	7
Recommendations based on total score	
Score ≥ 5: High risk	Consult urologyObtain testicular Doppler ultrasound
Score 2–4: Intermediate risk	Notify urologyObtain testicular Doppler ultrasound
Score 0–1: Low risk	Work up and disposition as per emergency department provider

*Testicular work up for ischemia and suspected torsion.

## METHODS

### Context

Our institution is a 296-bed tertiary care pediatric academic center located in Milwaukee, Wisconsin. It is the only children’s hospital in southeastern Wisconsin and is a primary referral site for surrounding urban and rural areas in Wisconsin, northern Illinois, and the upper peninsula of Michigan. In 2019, the hospital had 16,322 admissions and 70,866 ED visits. The ED is staffed by pediatric emergency medicine (PEM) physicians, PEM fellows, and advanced practice providers equipped to get TDUS in any patient with a concern for TT at any time of day or night. Pediatric urology fellows and faculty attend emergency urology consults 24 hours a day and 7 days a week using an on-call rather than an in-house model. Urology residents are expected to be at the bedside for all TT consults to schedule surgery immediately for TDUS confirmed cases of TT. In 2019, 33 patients underwent emergency scrotal exploration for TT; 22 were diagnosed in our ED.

### Interventions

**Fig. 1.**
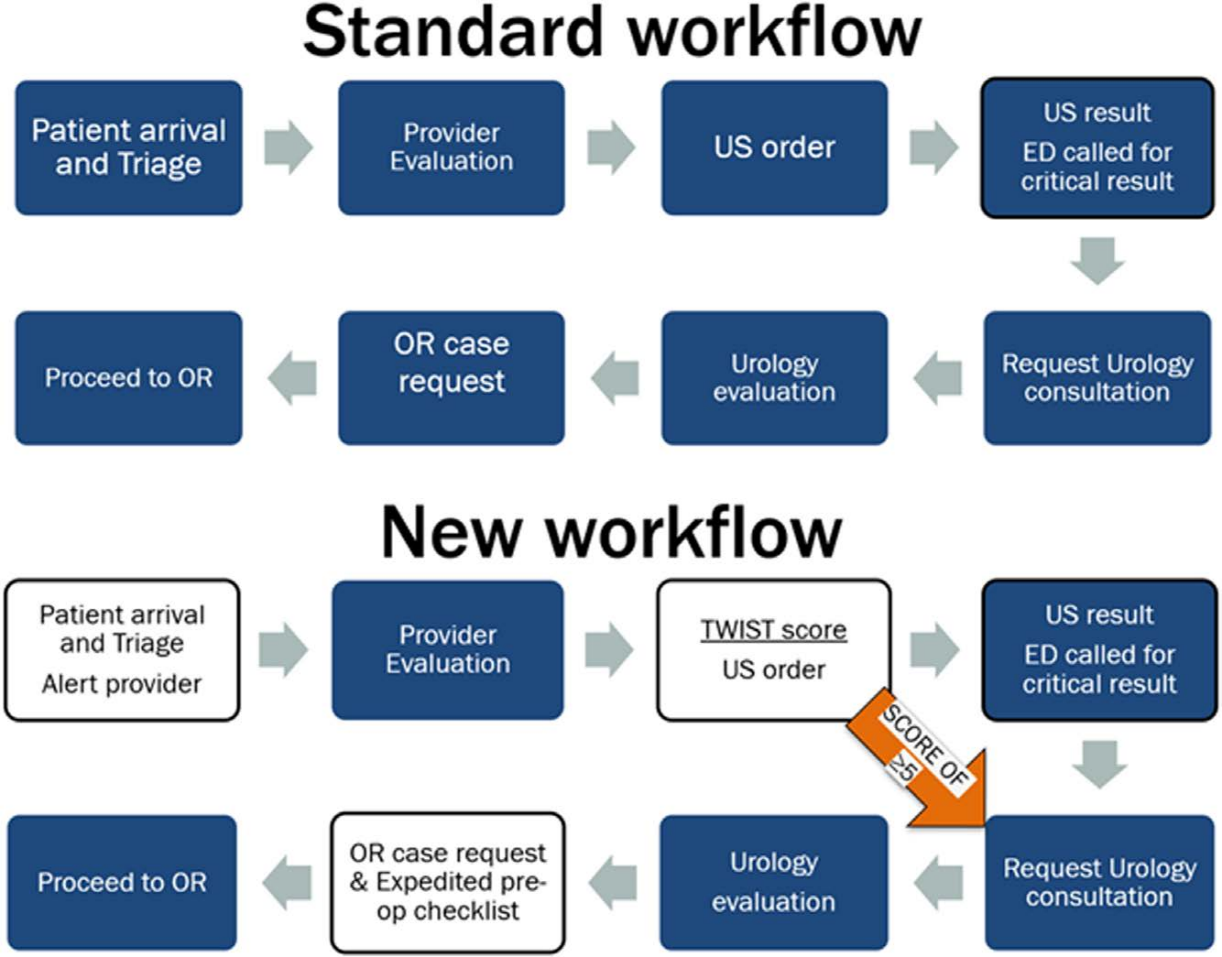
Process map comparing original workflow for TT workup compared with revised workflow. OR, operating room; TDUS, testicular doppler ultrasound.

**Fig. 2.**
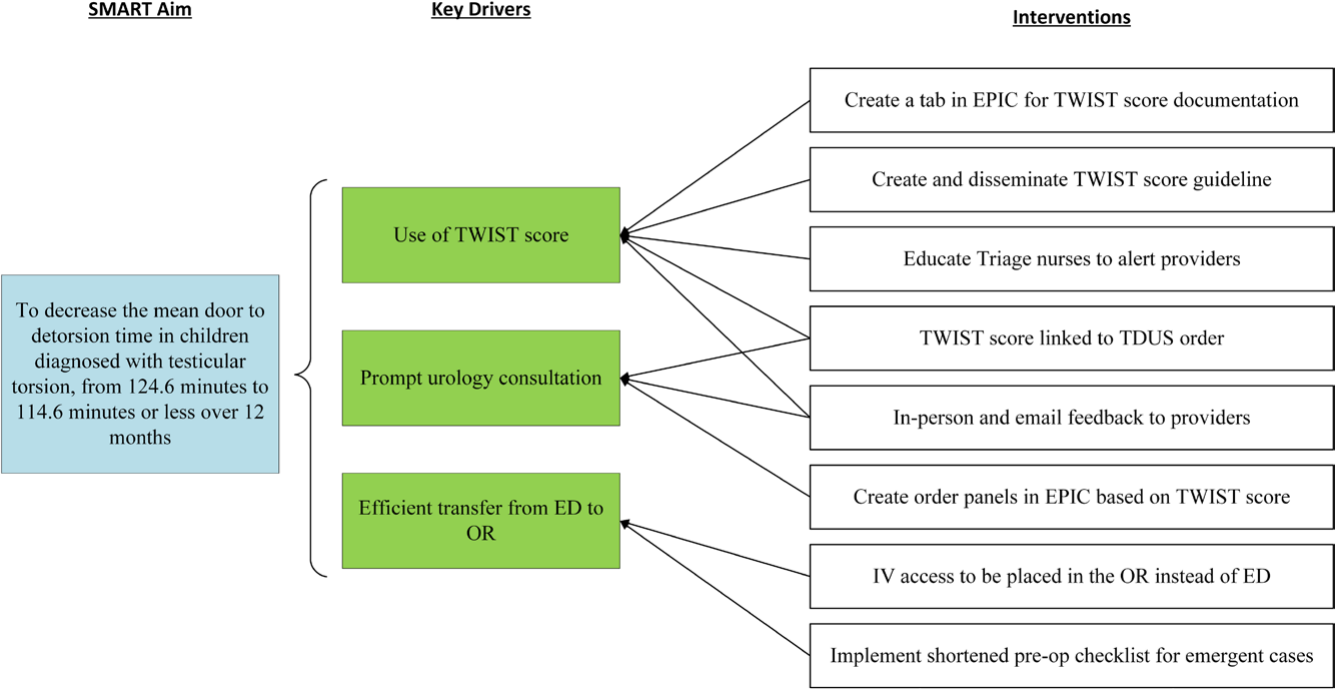
Key driver diagram. Epic refers to Epic electronic health record system. TWIST, testicular workup for ischemia and suspected torsion.

Patients presenting with a chief complaint of testicular pain or groin discomfort who underwent TDUS were included in the analysis. Patients who had a discharge diagnosis of testicular pain without TDUS were also included in the analysis to ensure compliance with TWIST score documentation. Patients with a confirmed diagnosis of TT before arrival were excluded. We established a multidisciplinary team including PEM physicians, urologists, a radiologist, and a nursing liaison. To better understand the ED flow of patients with potential TT, we created a process map from patient ED arrival to OR arrival (Fig. 1). OR arrival time was defined as when a patient was moved into the operating room (OR) and not the preoperative holding area. Using failure mode and effects analysis, we identified missed opportunities to identify potential cases during triage, opportunities for prompt urology consultation before the TDUS result, and identified steps to mitigate delays in ED to OR transfer. We aimed to improve communication and eliminate unnecessary steps with the new process flow. The key drivers to achieve our goal were as follows: (1) TWIST score use, (2) prompt urology consultation, and (3) efficient transfer from ED to OR (Fig. 2). We created the TWIST score guideline in collaboration with urology and PEM providers (Table 1). We incorporated a TWIST score tab in EPIC, allowing documentation as a part of typical provider workflow. We educated all ED nurses to identify any patient with a chief complaint of testicular pain and triage them as an ESI level 2 (high acuity) and immediately notify the ED charge attending or advanced practice providers. Providers rapidly examined these patients and documented the TWIST score to determine risk categorization and ordered TDUS based on the TWIST score or when there was a clinical concern for TT, such as a suspicious history but equivocal clinical findings. For patients with a TWIST score of 5 or above, the provider-initiated early urology consultation immediately by calling urology and discussing the case over the phone. With early consultation, urology residents were available in the ED, when TDUS confirmed the diagnosis to evaluate the patient immediately and initiate subsequent steps for surgery. We identified early urology consultation based on provider documentation of telephone conversation with urology immediately after placing Testicular Doppler ultrasound (TDUS) order and before the TDUS result. For patients with TWIST scores between 2 and 4, providers were advised to notify urology with a text message through the pager system. No phone discussion was required because some of the patients in this category were eventually diagnosed with TT. The radiology attending called the ED for all TDUS confirmed TT cases and documented the phone call time in the report, which we considered as TDUS result time. To expedite preparation and transition to OR, we reached agreement with the anesthesia team to accept patients to the OR without vascular access to avoid delays in ED to OR transfer and to utilize a shortened preop checklist (see table, Supplemental Digital Content 1, http://links.lww.com/PQ9/A385).

### Study of the Interventions

We monitored TWIST score documentation rate by reviewing an automated weekly report generated on all patients with a chief complaint of testicular pain, groin swelling, groin pain, or genitourinary (GU) problem. We set a goal to screen 80% or more of eligible patients using the TWIST score. The availability of the TWIST score tab in EPIC combined with provider and nursing education resulted in increased TWIST score documentation, but the documentation did not reach our 80% target. Upon further data review, we noted the TDUS order could serve as a point of intervention to remind providers about TWIST score documentation buttons in TDUS order. We created order panels in EPIC based on TWIST score to initiate early urology consultation and TDUS order for TWIST score of 5 or above.

Continued nursing education about the QI project and the interventions was maintained with the help of a nurse educator and a nurse champion. During daily huddles and monthly meetings, updates and feedback were provided to nurses. For the first 12 months, we reviewed the data biweekly and sent feedback via email to providers about TWIST score documentation and initiation of early urology consultation.

### Measures

The primary outcome measure was door-to-detorsion time. This was measured from the time patient arrived in the ED to the time patient arrived in the OR. The secondary outcome measure was orchiectomy rate. We identified patients who underwent orchiectomy by manual chart review. Our process measures were as follows: (1) TWIST score documentation rate which was the proportion of patients evaluated for TT who had a documented TWIST score, and (2) early urology consultation rate, defined as communication with urology immediately after placing TDUS order and before TDUS result, as documented by the ED provider. We tracked initiation of early urology consultation by manually reviewing the charts of patients diagnosed with TT and with a TWIST score of 5 or more. These process measures reflect compliance with our interventions on the patient population. TDUS utilization rate and urology consultation rate were the balancing measures to monitor resource utilization.

### Analysis

We used descriptive statistics to describe the patient population. Statistical process control charts were used to measure the impact of our interventions in real-time with control limits set at ±3 standard deviations. The centerline for the p chart used for TWIST score implementation was revised when 8 consecutive points were above or below the prior mean. A cumulative summary (CUSUM) chart was used to identify special cause variation for door-to-detorsion time because the small shifts in improvement were not identified by routine special cause criteria.^9^

### Ethical Considerations

Pursuant to Medical College of Wisconsin IRB policy, this project was deemed quality improvement/nonhuman subjects research and required no additional IRB review.

## RESULTS

**Fig. 3.**
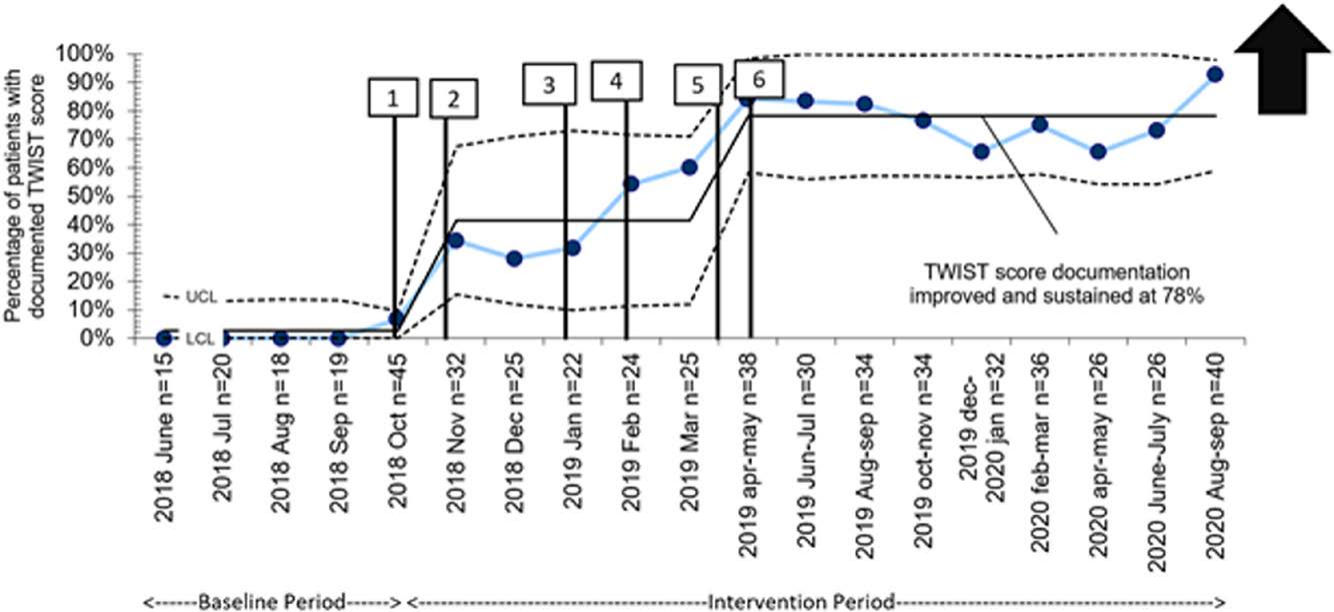
P chart showing the process measure “TWIST score documentation rate.” Each data point represents the percentage of patients with documented TWIST score for that time period; mean and control limits for the data are also shown. The following 6 interventions were sequentially shown on the graph corresponding to the time frame they were studied and introduced: (1) TWIST score tab created in EPIC; (2) Small group discussion to prepare guideline; (3) TWIST score guideline shared with all providers for review; (4) TWIST score guideline finalized and available for everyone in EPIC for reference; (5) TWIST score linked to TDUS order and created order panels in EPIC; (6) RN engagement and personal communication to providers. Arrow pointing upwards represents the goal to increase the outcome over time. TWIST, testicular workup for ischemia and suspected torsion.

Our baseline period was from January 2017 to September 2018, and the intervention period was from October 2018 to September 2020. The demographics of patients evaluated for TT and diagnosed with TT are presented in Table 2. There were no differences in the population between baseline and intervention periods. During the intervention period, 816 patients were evaluated for TT, 736 (90%) patients underwent TDUS. Of the 736 patients who received TDUS, 46 (6%) patients were diagnosed with TT. The TWIST score utilization rate improved from 0% to 78% over the first 6 months of the intervention period and was sustained at that rate for 18 months after that (Fig. 3). Among the 46 patients with TT, a TWIST score was documented in 36, of which 31 had a score of 5 or more (86%); 4 patients had a score of 4 (11%) and 1 patient had a score of 3 (3%).

**Table 2.** Demographics

	Preintervention(January 2017–September 2018)	Postintervention(October 2018–September 2020)	*P*
**Entire cohort**			
n	616	816	
Age in years, mean (SD)	8.9 (5.6)	8.6 (6.0)	0.163^*^
Race, %			0.324^†^
African American	26.3	28.5	
White	62.2	62.2	
Other	11.5	9.3	
TDUS utilization rate, %	72.3	67.8	0.277^†^
**Patients diagnosed with testicular torsion**			
N	31	46	
Orchiectomy rate	25.8% (8/31)	23.9% (11/46)	0.850^*^
Age in years, mean (SD)	13.5 (3.7)	13 (3.5)	0.306^†^
Race, %			0.603^†^
African American	35.5	37.0	
White	48.4	54.3	
Other	16.1	8.7	

^*^Mann-Whitney U test.

^†^Chi-square test.

**Fig. 4.**
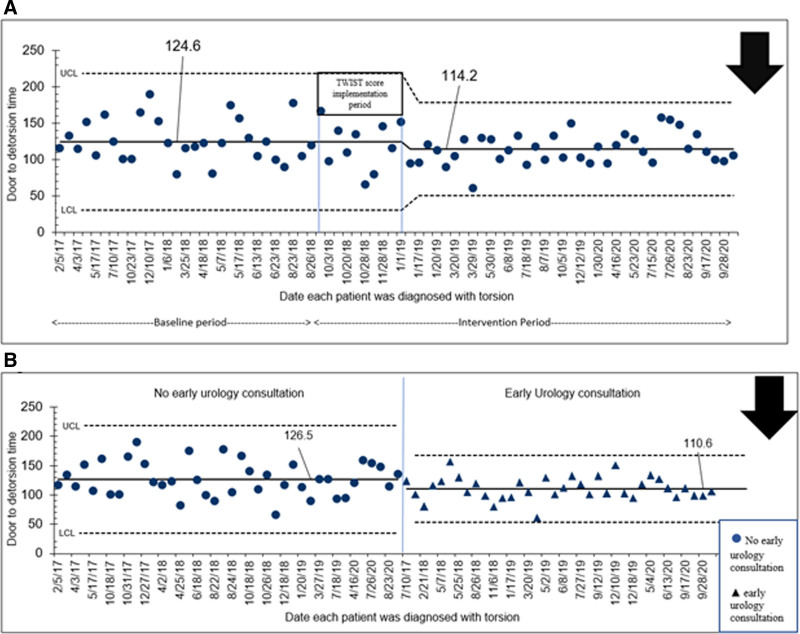
I-chart showing door-to-detorsion time (in min) for patients diagnosed with testicular torsion. A, Each patient shown sequentially. Centerline and control limits were revised based on the shift identified using CUSUM chart (provided as figure, Supplemental Digital Content, http://links.lww.com/PQ9/A385). B, Patients with and without early urology consultation displayed side by side in 2 separate groups. Arrow pointing downward in each figure represents the goal to decrease the outcome over time. ● represents patient who had no early urology consultation and ▲ represents patient who had early urology consultation.

The mean door-to-detorsion times improved from 124.6 to 114.2 minutes (Fig. 4A). The shift in the door-to-detorsion time was identified using CUSUM chart (see figure, Supplemental Digital Content, http://links.lww.com/PQ9/A385). We stratified our population based on the initiation of early urology consultation. We noted that the mean door-to-detorsion time was 15 minutes shorter in patients who had initiation of early urology consultation (Fig. 4B). Since implementing the TWIST score, the early urology consultation rate improved to 60% (27 out of 45 cases) compared with 40% (8 out of 20 cases) before implementation. The improvement in orchiectomy rate from 25.8% to 23.9% was not statistically significant.

TDUS rate decreased from 72.3% to 67.8% and was not statistically significant. In addition to the patients diagnosed with TT, early urology consultation triggered by a TWIST score of 5 or more was found in 15% (8 patients) who were not diagnosed with TT.

## DISCUSSION

### Summary

Successful implementation of the TWIST score as a tool to increase early urology consultation for patients with concern for TT resulted in improvement in the door-to-detorsion times. Our process improvement did not translate to reduction in orchiectomy rate. Multidisciplinary collaborative work between urology, radiology, and emergency medicine was the key strength of this QI project.

### Interpretation

Gold et al^5^ attempted to understand the role of door-to-detorsion time in their population with 51.1% orchiectomy rate and estimated that every 10 minutes of delay in the ED increased the chance of nonviable testis by 4.8%. Despite our 10 minutes reduction in door-to-detorsion time, we only saw a modest decrease of 1.9% in orchiectomy rate, which was not statistically significant. Others including Zee et al^10^ noted substantial improvements in door-to-detorsion time (from 196 to 127 min) but failed to reduce the orchiectomy rate from 24%. Although decreasing orchiectomy rates is the gold standard of timely care for testis torsion, orchiectomy is a complex outcome that is influenced by factors that occur outside of the hospital setting, such as time between onset of symptoms and presentation to a healthcare facility. Our expedited pathway holds promise for any healthcare facility with high orchiectomy rates and lack of in-house urology coverage but may have a lower limit beyond which orchiectomy rates do not improve without additional community education and engagement efforts which are outside of the scope of our current study.

The TWIST score was originally intended to decrease the number of patients who needed TDUS to diagnose TT^6^ but we used it differently. Since previous studies reported diagnosis of TT associated with TWIST scores of 0–4,^7,8^ we did not use TWIST score to limit the use of TDUS. Repeated email and in-person feedback to providers about their performance, linking of TWIST score to TDUS order, and creation of TWIST order panels were the key interventions that were particularly effective. One of the key strengths of the TWIST score is the increasing positive predictive value with higher scores. This is the first study to utilize the TWIST score as a screening tool to identify patients at high risk for TT to expedite care to the OR. A TWIST score of 7 has 100% positive predictive value for TT in our population and is consistent with previous studies.^7,8^

Over 2 years, a TWIST score of 5 and above had an 86% positive predictive value and a false positive rate of 4.5% (13 out of 291). None of the patients with a TWIST score of 0–2 were diagnosed with TT. We missed the opportunity to document TWIST scores in 20% of patients for which providers ordered TDUS with a concern for torsion. During busy times of the day, when patient care rooms were not immediately available or due to a lack of provider ability to immediately assess new patients, a TDUS was ordered before provider examination and TWIST score documentation. TWIST score documentation was strongly recommended but not a mandatory step in our QI project because of concerns of a possible delay in care for some patients. Early urology consultation was missed in 21.7% (10 patients) diagnosed with TT, of which 15% (7 patients) had a TWIST score of 5 or more. We will continue offering feedback to providers to decrease missed opportunities. Unintended consequences from our study include an increase in urology consultation based on TWIST score of 5 in 15% (8 patients). None of the patients with TWIST score of 0, 1, or 2 were diagnosed with TT in our study, and all the patients with a TWIST score of 7 were diagnosed with TT.

Our next steps include working with urology and OR team to decrease variation in the care from ED departure to OR arrival time and decrease the TDUS utilization rate based on the TWIST score. We will sustain the improvements from this QI project by periodically monitoring our outcome measures to identify any deviations from the interventions implemented in the QI project and provide feedback to providers and nurses.

Although our TDUS utilization rate seems high, we did not find published data to compare the TDUS rate in evaluating acute scrotum. Testicular doppler ultrasound (TDUS) is an adjuvant imaging study with high diagnostic accuracy for TT and is frequently utilized to decrease the likelihood of negative scrotal exploration.^4^ This practice is beneficial in clinical settings where US is immediately available and would not delay surgical exploration. In resource-limited settings where US is not readily available and would delay surgical exploration, it is common practice to omit imaging in cases of high likelihood of torsion.

### Limitations

The factors that limit the generalizability of our study are reliance on manual chart review, single-center experience, education-based interventions, reliance on before and after analysis, access to data, and EPIC analyst. Previous studies have shown a relationship between the duration of testicular ischemia and orchiectomy rate, but we do not have accurate data on symptom duration in our patient population.^3,4^ Early urology consultation was expected to improve the availability of urology resident at the bedside, but we could not establish metrics to report patient evaluation time by urology residents. All the interventions in our QI project were directed at processes in the ED. We presumed that most patients would arrive in OR within 10 minutes after departure from the ED. But 52% (24 patients) needed more than 10 minutes with a range of 12−68 minutes. This contributed to longer than anticipated door-to-detorsion time and were attributed to OR workflow. As we did not have OR staff as part of the team, we could not identify specific reasons for these delays. We could not study the value of “notifying urology” with a text page about patients with TWIST scores 2–4 as we could not track and analyze these data. Additionally, our institutional practice requiring TDUS confirmation of TT before scheduling OR could limit the generalizability of this QI project.

### Concluding Summary

We successfully implemented a collaborative QI project to expedite the ED care of patients with suspected testicular torsion. Following our interventions, we saw significant improvements in door-to-detorsion time with a decrease in variation in care between patients. Elements of this model could be applied to other surgical conditions that require early recognition and expedited ED to OR transfer.

## DISCLOSURE

The authors have no financial interest to declare in relation to the content of this article.

## ACKNOWLEDGMENTS

Assistance with the study: We thank the faculty of I2S2 and AIM courses at Cincinnati Children’s Hospital, and Ashley Servi RN, CNS at Children’s Wisconsin.

## Supplementary Material


