# Supplementary material for: Quality Improvement Project to Improve the Timeliness of Care for Children With Testicular Torsion in the Emergency Department
Source: Pediatr Qual Saf. 2022 Jul 18;7(4):e576. doi: 10.1097/pq9.0000000000000576 (PMC10997231; doi:10.1097/pq9.0000000000000576)
Supplement: Supplementary file 1 [file pqs-7-e576-s001.pdf]

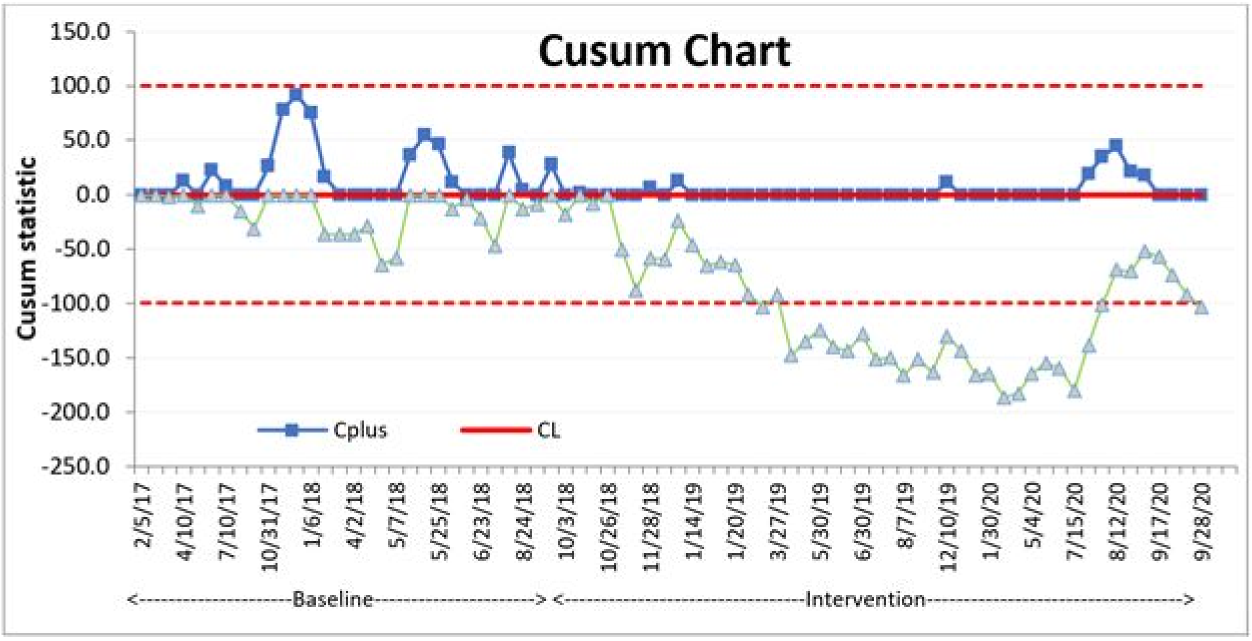
Cusum Chart
Cusum statistic
150.0
100.0
50.0
0.0
-50.0
-100.0
-150.0
-200.0
-250.0
Cplus
CL
2/5/17
4/10/17
7/10/17
10/31/17
1/6/18
4/2/18
5/7/18
5/25/18
6/23/18
8/24/18
10/3/18
10/26/18
11/28/18
1/14/19
1/20/19
3/27/19
5/30/19
6/30/19
8/7/19
12/10/19
1/30/20
5/4/20
7/15/20
8/12/20
9/17/20
9/28/20
<--------------------Baseline---------------------> <------------------------------Intervention----------------------------------->

SDC, Table 1: Preop checklist used at Children's Wisconsin. For patients who qualify for 'shortened preop checklist, only the items highlighted in peach are collected

**Height/Weight**
Height Current Within 7 Days
Weight Current From Today
**ID Bands On and Verified**
ID Band(s) Applied/Verified
Other Bands (Allergies/Fall Risk/Etc.) Applied/Verified
**Pre-Procedure Blood Products**
Blood Band(s) Applied/Verified
Blood Products Ordered
ABO/Rh Result (read only)
Crossmatch Exp. (read only)
**NPO**
Date of Last Solid Meal
Time of Last Solid Meal
Date of Last Milk, Formula, or Breast Milk with Additives – PO/GT/NG
Time of Last Milk, Formula, or Breast Milk with Additives – PO/GT/NG
Date of Last Breast Milk – PO/GT/NG
Time of Last Breast Milk – PO/GT/NG
Date of Last Clear Liquid or All J-tube Feeds
Time of Last Clear Liquid or All J-tube Feeds
**Last Void**
Date of Last Void/ Wet Diaper Prior to Procedure
Time of Last Void/ Wet Diaper Prior to Procedure
**Pre-Procedure Bath**
Soap/H2O Head-to-Toe Bath Night Before
CHG Bath Night Before Procedure
CHG Bath Day of Procedure
**Pre-Procedure Safety**
Site Marked *SURGICENTER ONLY*
COVID Test Completed
Jewelry Removed
**Additional Important Information**
Additional Important Information
Pre-Procedure Verification
Surgical/Procedure Consent Signed

History and Physical Completed
Pre-Procedure Lab/Tests Completed
SCDs Applied
Sickle Cell Screening Status
Family Contact Information (During Procedure)
Family Contact Number
Family Waiting Location
Family Waiting Area Room
Family Waiting Area B Room
Family Waiting Area C Room
Family Waiting Area D Room
Pre-Procedure Medications
Pre-Procedure Medications Given
Patient Preparation
Undergarments (ALL) Removed
Hair Free of Accessories
Nail Polish Removed
Belongings That Remain With Patient
Patient Belongings
Transfer of Nursing Care (Pre-Procedure)
Patient Sent To
Accompanied By (Comment for name as needed)
Contact for Pre-Procedure Handoff – RN Name
Contact for Pre-Procedure Handoff – RN Phone
Safe Environment
ID/ Risk Bands On and Verified
